# Overnight sleeping heart rate variability of Army recruits during a 12-week basic military training course

**DOI:** 10.1007/s00421-022-04987-3

**Published:** 2022-07-14

**Authors:** Michael J. Macartney, Penelope Larsen, Neil Gibson, Scott Michael, Jace Drain, Gregory E. Peoples, Herbert Groeller

**Affiliations:** 1 Centre for Medical and Exercise Physiology, Faculty of Science, Medicine and Health, University of Wollongong, Wollongong, NSW 2522 Australia; 2 School of Clinical Medicine, Faculty of Medicine and Health, University of New South Wales, Sydney, NSW 2444 Australia; 3 Defence Science and Technology Group, Fishermans Bend, VIC 3207 Australia; 4 Graduate School of Medicine, Faculty of Science, Medicine and Health, University of Wollongong, Wollongong, NSW 2522 Australia; 5 School of Medical, Indigenous and Health Sciences, Faculty of Science, Medicine and Health, University of Wollongong, Wollongong, NSW 2522 Australia

**Keywords:** Cardiorespiratory fitness, Parasympathetic activity, Vagal activity, Military, Exercise

## Abstract

**Purpose:**

This study aimed to quantify sleeping heart rate (HR) and HR variability (HRV) alongside circulating tumor necrosis factor alpha (TNFα) concentrations during 12-week Basic Military Training (BMT). We hypothesised that, despite a high allostatic load, BMT would increase cardiorespiratory fitness and HRV, while lowering both sleeping HR and TNFα in young healthy recruits.

**Methods:**

Sixty-three recruits (18–43 years) undertook ≥ 2 overnight cardiac frequency recordings in weeks 1, 8 and 12 of BMT with 4 h of beat-to-beat HR collected between 00:00 and 06:00 h on each night. Beat-to-beat data were used to derive HR and HRV metrics which were analysed as weekly averages (totalling 8 h). A fasted morning blood sample was collected in the equivalent weeks for the measurement of circulating TNFα concentrations and predicted VO_2_max was assessed in weeks 2 and 8.

**Results:**

Predicted VO_2_max was significantly increased at week 8 (+ 3.3 ± 2.6 mL kg^−1^ min^−1^; *p* < 0.001). Sleeping HR (wk1, 63 ± 7 b min^−1^) was progressively reduced throughout BMT (wk8, 58 ± 6; wk12, 55 ± 6 b min^−1^; *p* < 0.01). Sleeping HRV reflected by the root mean square of successive differences (RMSSD; wk1, 86 ± 50 ms) was progressively increased (wk8, 98 ± 50; wk12, 106 ± 52 ms; *p* < 0.01). Fasted circulating TNFα (wk1, 9.1 ± 2.8 pg/mL) remained unchanged at wk8 (8.9 ± 2.5 pg/mL; *p* = 0.79) but were significantly reduced at wk12 (8.0 ± 2.4 pg/mL; *p* < 0.01).

**Conclusion:**

Increased predicted VO_2_max, HRV and reduced HR during overnight sleep are reflective of typical cardiorespiratory endurance training responses. These results indicate that recruits are achieving cardiovascular health benefits despite the high allostatic load associated with the 12-week BMT.

## Introduction

Measurement of heart rate (HR) and heart rate variability (HRV) responses are commonly employed as non-invasive surrogate assessments of cardiac autonomic modulation (Michael et al. 2017; Task Force 1996), often used to inform exercise training programs (Singh et al. 2018). Compared with untrained individuals, participation in cardiorespiratory endurance training has been associated with lower resting HR and increased HRV, indicative of stronger cardiac parasympathetic (vagal) autonomic modulation (Goldsmith et al. 1992; Seals and Chase 1985). In contrast, studies investigating exposure to prolonged high-volume cardiorespiratory endurance training demands or stressors such as compromised sleep, insufficient recovery, and fatigue often demonstrate reduced HRV, although findings are inconsistent (Meeusen et al. 2013; Westphal et al. 2021; Pichot et al. 2002). It is becoming increasingly recognised that HRV responses are dependent on a range of physiological and environmental factors which are difficult to control in applied research settings (Fatisson et al. 2016), likely influencing previous study outcomes.

The Australian Army Basic Military Training (BMT) program exposes a relatively homogenous group of recruits to controlled nutrition, psychological, physical,and military skills training designed to improve physical function and develop foundational military skills (Drain et al. 2017). An important component of the Australian Army BMT is the physical training program which consists of approximately 40 sessions. The focus of the physical training program is higher-intensity, lower-volume strength, and cardiorespiratory endurance training, with ~ 93% of sessions completed over the first 9 weeks of BMT on-barracks (Burley et al. 2020), before the 2-week field training phase. In addition to structured physical training and recovery, all participants are exposed to a prescribed sleep opportunity (‘lights out’ at 22:00 h and ‘wake up’ at 06:00 h) when in barracks. However, despite the primary objective of BMT, the physical and mental challenges when combined with inadequate periods of recovery lead to high allostatic stress that can undermine the rate of physiological adaptation (Drain et al. 2017). Accordingly, BMT offers a uniquely controlled setting in which to investigate HR and HRV responses to prolonged physical training and stress.

Previously, the root mean square of successive differences (RMSSD) of HR, a common HRV estimate of cardiac vagal autonomic modulation (Task Force 1996), has been demonstrated to increase following BMT (Grant et al. 2016) and more advanced military training courses (Jouanin et al. 2004) when analysed using waking short-term (5 min) recordings. While short-term and longer-term (e.g., 24 h) HRV recordings are widely used in non-clinical and clinical investigations, consideration needs to be given to the settings in which data are collected when interpreting HR and HRV (Kleiger et al. 2005), particularly in military populations (Hinde et al. 2021). For example, independent of environmental and behavioural changes, circadian rhythm influences cardiac autonomic modulation of HR to produce typical diurnal variability, whereby nadir occurs overnight alongside increased HRV, reflective of increased vagal autonomic activity (Furlan et al. 1990; Task Force 1996). Furthermore, night sleep is an important period for both physiological and psychological recovery (Brosschot et al. 2007) and HRV recording reliability may be enhanced with reduced influence from environmental factors (Pichot et al. 2000). Taking these issues into consideration, this study aimed to monitor HR and HRV during prescribed sleep opportunities (22:00–06:00 h) over the course of BMT as a practical and standardised approach in an applied military setting where day-to-day activities can vary substantially.

In addition to enhance cardiorespiratory fitness and cardiac physiological adaptations, participation in physical activity or exercise training is associated with a reduction in systemic inflammation (Lavie et al. 2011). However, the changes in pro-inflammatory cytokine markers, such as tumor necrosis factor α (TNFα), during and in response to BMT have been inconsistent, with several studies observing an increase (Booth et al. 2006; Gomez-Merino et al. 2003; Ojanen et al. 2018) while a more recent study has shown a decrease in TNFα at the completion of BMT (Tait et al. 2021). Recent evidence postulates that an increase in vagal nerve activity, which shares innervation to the gut and the spleen, may indirectly inhibit the release of circulating TNFα (Bonaz et al. 2016) and other specific inflammatory markers (Alen et al. 2021). Considering this postulation, increased HRV observed during BMT (Grant et al. 2016) could be further associated with a reduction in TNFα. However, the relationship between HRV, as a reflection of cardiac vagal modulation, and TNFα has yet to be investigated in young healthy adults.

The principle aim of this investigation was to quantify sleeping HR and the well-established vagally mediated HRV metric of RMSSD in recruits, during prescribed overnight sleep opportunities, throughout BMT. The primary hypothesis was that overnight sleeping HR would be reduced and RMSSD would be increased alongside improvements in cardiorespiratory fitness as a result of 12-week BMT. A secondary aim was to examine the association between fasted morning circulating TNFα concentrations and vagally mediated RMSSD. Finally, a number of less-established HRV metrics were included for exploratory analyses.

## Materials and methods

### Experimental overview

This study collected data during a 12-week BMT program and was part of a larger project designed to quantify training loads and monitor the physiological and psychological responses of recruits during BMT, the experimental procedures and results of which have been published previously (Larsen et al. 2022). All recruits completed identical training syllabuses that included orientation, progressive physical training (40 sessions) and military skills (weeks 1–9), field training (weeks 10–11), and ceremonial preparations and March out (week 12) as previously described (Larsen et al. 2022). The typical training day (06:00–22:00 h) in the first nine weeks included supervised physical training and military education sessions in that order. Week 1 represents baseline data, week 8 represents the end of prescribed physical training and week 12 represents the end of BMT. Overnight cardiac frequency monitoring was initiated in week 1 following the provision of consent and recruits’ initial medical assessment. For week 12, data were recorded across 3 nights due to preparation for the march out parade. In addition, finger prick blood samples were collected in a fasted state upon wakening at the commencement of BMT (week 1), week 8 and week 12 of training to assess changes in circulating TNFα concentrations. Cardiorespiratory physical fitness was assessed via multi-stage shuttle test performance during weeks 2 and 8 of BMT.

### Participants

One hundred male and female recruits (18–43 years) volunteered to participate in the study in which 63 recruits (males: *n* = 55, females *n* = 8; 23 ± 5 years; 76.8 ± 14.0 kg; 24.3 ± 3.4 kg m^−2^) met the inclusion criteria; i.e., ≥ 2 overnight sleep recordings with a minimum 4 h of continuous RR-interval data collected between 00:00 and 06:00 h in week 1, 8 and 12 of BMT, and completed BMT within the scheduled 12 weeks. The remaining 37 recruits were excluded from the current study due to withdrawal of consent, delayed training schedule (e.g., injury or inability to meet training standards), discharged from training, or failure to meet inclusion criteria. Recruits were from three platoons that commenced training in August and September, 2019, at the Army Recruit Training Centre, Kapooka (Australia). The study procedures and risks were explained verbally and provided in writing to each recruit before they provided written informed consent. The study was approved by the Department of Defence and Veterans’ Affairs Human Research Ethics Committee (protocol number: 083-18).

### Heart rate and HRV measurement

Overnight cardiac frequency was recorded using electrocardiographic chest-strap HR monitors, with the RR-interval data recordings downloaded from the devices and imported to the Polar Team2 System (Polar Electro Oy, Kempele, Finland). The RR-intervals were then exported into Kubios software for subsequent analysis (Tarvainen et al. 2014). All files were passed through an automatic noise detection filter and artefact correction algorithm (Lipponen and Tarvainen 2019) which removed artefacts due to ectopic beats, missed beats etc. Artefacts were replaced using cubic spline interpolation leaving only physiologically normal-to-normal interval (NN-interval) time series before HRV analysis was completed. With the use of the NN-interval time series of each participant, absolute HR and HRV data were calculated as weekly averages using a 4-h window collected between 24:00 and 06:00 h from the same two nights (totaling 8 h from each weekly time point). In the majority of participants, data was collected from 24:00 to 04:00 to reduce the influence of the initial stages of sleep and awakening. When a file had > 5% of the total beats corrected (AVG, 1.1%; SD, ± 0.9%), visual evidence of sleep disturbances or an incomplete overnight recording that prohibited analysis of a 4-h window, it was not included in the analysis and replaced with a file from the night before/after.

HRV metrics were computed using time, frequency and non-linear domains. The study investigated RMSSD of NN-intervals as the most established HRV metric reflecting vagal modulation of HR (Task Force 1996). RMSSD was also investigated following correction for HR—by division of RMSSD by average NN-interval to the power 4 (Sacha et al. 2013). In addition, the low frequency (LF, 0.04–0.15 Hz), high frequency (HF, 0.15–0.4 Hz), LF/HF ratio, sample entropy (SampEn), approximate entropy (ApEn) along with both the short-term detrended fluctuation analysis alpha-1 (DFAα1) and long-term alpha-2 (DFAα2) scaling exponents were included for further exploratory analyses. Power spectra were calculated by means of fast Fourier transformation. The European Heart Rhythm Association (EHRA) HRV signal analysis position paper provides further clarification of the HRV metrics reported in this study (Sassi et al. 2015).

### Tumor necrosis factor α (TNFα) measurement

Blood sample collection was performed using the finger prick method in a fasting state (between 06:00 and 07:00 h). In brief, the hand of each recruit was warmed using gloves and the finger was cleaned using an alcohol swab. A 21-G lancet (Sarstedt, Germany) puncture enabled the finger to bleed freely and enough volume was collected to fill a 300 µL tube treated with lithium heparin (Sarstedt, Germany). Samples were centrifuged at 4000 rpm for 5 min at 4 °C. Plasma obtained was stored at − 80 °C and later analysed independently by a registered laboratory. Samples were analysed in duplicate using a High Sensitive T Cell Cytokine Panel (HSTCMAG-28SK, Milliplex, Merck) to provide raw values for circulating TNFα. Results dropping below the detection threshold were allocated the value for the assay’s minimum detectable limit (TNFα: 0.16 pg/mL). Analyses had an intra-assay percent coefficient of variation (%CV) of < 6% and an inter-assay %CV of < 20%.

### Cardiorespiratory physical fitness

Cardiorespiratory physical fitness was assessed via multi-stage shuttle test performance during weeks 2 and 8 of BMT. Performance scores in the multi-stage shuttle test were used to predict VO_2_max according to Ramsbottom et al. (1988).

### Statistical analysis

Prior to statistical analysis, the distribution of continuous data was analysed for normality using the D’Agostino–Pearson omnibus (K2) test and homogeneity of variances was confirmed via Brown–Forsythe test. As RMSSD was not normally distributed, natural logarithmic transformed observations were analysed, and these were normally distributed. A paired *t* test was used to compare differences in VO_2_max at week 2 and week 8. A repeated measures one-way analysis of variance (ANOVA) was used to compare differences between BMT weeks for HR and HRV metrics recorded during overnight sleep. Tukey’s post hoc tests were applied to identify where any significant differences occurred. A mixed-effects model was used to compare differences between BMT weeks for circulating TNFα concentrations and Pearson’s correlation analysis was used to investigate associations with RMSSD in a subset of the recruits at weeks 1 (*n* = 50), 8 (*n* = 28) and 12 (*n* = 39). Data are presented as mean (95% CI) unless otherwise stated and statistical significance was accepted at *p* ≤ 0.05. Statistical analyses were completed in GraphPad Prism (v9, GraphPad Software, USA).

## Results

### Cardiorespiratory physical fitness

At week 2, predicted VO_2_max of participants was 42.5 ± 3.8 (mL kg^−1^ min^−1^). The multi-stage shuttle test conducted at weeks 2 and 8 demonstrated a significant increase in predicted VO_2_max (+ 3.3 ± 2.6 mL kg^−1^ min^−1^; *p* < 0.001).

### Heart rate, RMSSD and TNFα

**Fig. 1:**
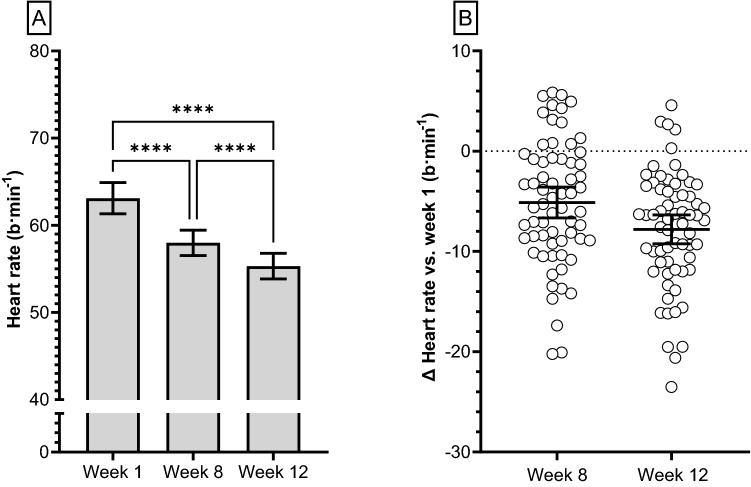
**a** Time-course changes in group mean (± 95% CI) overnight sleeping heart rate collected in participants (*n* = 63) during 12-week Basic Military Training. **b** Changes in overnight sleeping heart rate from week 1 (dashed line). Each individual response is plotted along with the group mean ± 95% CI (solid black line). Data analysed using a one-way repeated measures ANOVA with Tukey’s multiple comparisons test. ****p < 0.0001. b min^−1^, beats per minute

**Fig. 2:**
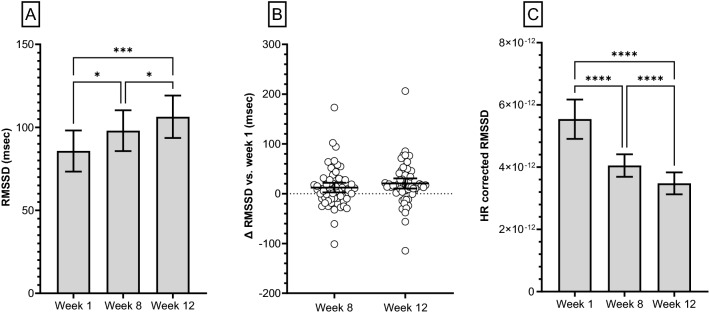
**a** Time-course changes in group mean (± 95% CI) overnight sleeping RMSSD collected in participants (*n* = 63) during 12-week Basic Military Training. **b** Changes in overnight sleeping RMSSD from week 1 (dashed line). Each individual response is plotted along with the group mean ± 95% CI (solid black line). **c** Time-course changes in group mean (± 95% CI) HR corrected overnight sleeping RMSSD. RMSSD is presented using msec for ease of physiological interpretation but was analysed after natural logarithmic transformation to achieve normal distribution. Data analysed using a one-way repeated measures ANOVA with Tukey’s multiple comparisons test. ****p < 0.0001; ***p < 0.001; *p < 0.05. *RMSSD*, root mean square of successive differences; *HR*, heart rate

**Fig. 3:**
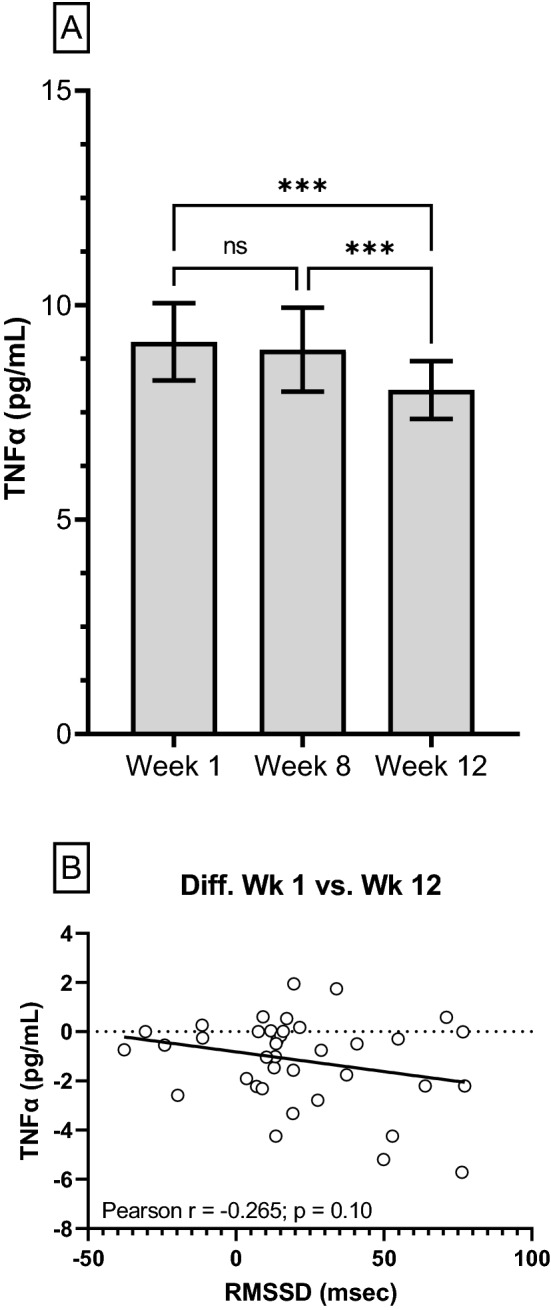
**a** Time-course changes in group mean (± 95% CI) circulating morning TNFα concentrations collected in participants (week 1: *n* = 50; week 8: *n* = 28; week 12: *n* = 39) during 12-week Basic Military Training. Data analysed using a mixed-effects model with Tukey’s multiple comparisons test. ***p < 0.001; ns, not significant. **b** Correlation between change in RMSSD and TNFα between week 1 and 12 of Basic Military Training in a subset of the recruits. Each individual response is plotted on the graph and a simple linear regression line of best fit (solid black line) has been fitted to the data with Pearson r and two-tailed p value displayed. *RMSSD*, root mean square of successive differences; *TNFα*, tumor necrosis factor alpha

The average overnight sleeping HR progressively reduced (*p* < 0.001) throughout BMT (Fig. 1a, b). More specifically, average overnight sleeping HR was 5 b min^−1^ slower at week 8, relative to week 1 and further reduced by 3 b min^−1^ at week 12, relative to week 8. Average overnight sleeping RMSSD progressively increased (*p* < 0.001) throughout BMT (Fig. 2a, b); however, when RMSSD was corrected for underlying HR it was observed to be progressively lower (Fig. 2c). Circulating TNFα concentrations remained unchanged at week 8 but were significantly reduced at week 12 (Fig. 3a). No correlations were observed between RMSSD and TNFα at week 1 (Pearson *r* = − 0.022; *p* = 0.88), week 8 (Pearson *r* = 0.021; *p* = 0.92) or week 12 (Fig. 3b; Pearson *r* = − 0.019; *p* = 0.25). Likewise, no evidence of a relationship was observed when RMSSD and TNFα were assessed as a relative change between weeks 1 and 8 (Pearson *r* = − 0.314; *p* = 0.11), 1 and 12 (Pearson *r* = − 0.265; *p* = 0.10) or between weeks 8 and 12 (Pearson *r* = 0.292; *p* = 0.12).

### Other HRV metrics

Frequency domain LF and HF power during week 8 remained unchanged from week 1. However, LF and HF power had increased significantly (*p* < 0.001) during week 12, relative to both week 1 and week 8 (Table 1). The average overnight sleeping LF:HF power ratio was significantly (*p* < 0.001) reduced at weeks 8 and 12 compared to week 1, with no differences observed between weeks 8 and 12 (Table 1). Relative to week 1, the average overnight sleeping non-linear domain HRV metrics ApEn and SampEn were significantly (*p* < 0.001) increased at weeks 8 and 12, with no further differences observed between weeks 8 and 12 (Table 1). Finally, the average overnight sleeping DFAα1 and α2 were significantly (*p* < 0.001) reduced at both week 8s and 12 compared to week 1 but no further significant decreases were observed between weeks 8 and 12 (Table 1).

**Table 1:** Overnight sleeping heart rate variability metrics collected in recruits during 12-week Basic Military Training

Variable, unit	Training Week			ANOVA for repeated measures			
	Week 1	Week 8	Week 12	Within subject		Between weeks	
	Mean [95% CI]	Mean [95% CI]	Mean [95% CI]	*F*	*P*	*F*	*P*
*Frequency domain*							
LF, msec^2^	2855 [2266, 3444]	3000 [2495, 3504]	3465 [2899, 4030]^a,b^	15.34	< 0.001	7.63	0.002
HF, msec^2^	3511 [2462, 4561]	4138 [3042, 5235]	4760 [3495, 6025]^a,b^	9.07	< 0.001	4.41	0.027
LF:HF	1.30 [1.12, 1.47]	1.12 [0.94, 1.30]^a^	1.09 [0.92, 1.26]^a^	18.65	< 0.001	11.12	0.001
*Non-linear domain*							
DFA α1	0.93 [0.88, 0.97]	0.86 [0.81, 0.90]^a^	0.84 [0.79, 0.88]^a^	12.80	< 0.001	23.11	< 0.001
DFA α2	0.34 [0.32, 0.36]	0.32 [0.31, 0.34]^a^	0.32 [0.30, 0.33]^a^	4.96	< 0.001	12.08	< 0.001
ApEn, bits	1.40 [1.37, 1.42]	1.42 [1.40, 1.44]^a^	1.43 [1.41, 1.45]^a^	9.89	< 0.001	10.96	< 0.001
SampEn, bits	1.53 [1.49, 1.57]	1.59 [1.55, 1.63]^a^	1.59 [1.56, 1.62]^a^	9.17	< 0.001	13.30	< 0.001

Data reported (*n* = 63). ^a^Significantly different from Week 1, ^b^Significantly different from week 8, *p* < 0.05 (repeated measures ANOVA with Tukey’s multiple comparisons test). RMSSD, root-mean-square of successive differences of NN-intervals; LF, low frequency power (0.04–0.15 Hz); HF, high frequency power (0.15–0.4 Hz); DFA α1, Detrended fluctuation analysis short-term exponent alpha 1; DFA α2, detrended fluctuation analysis long-term exponent alpha 2; ApEn, approximate entropy; SampEn, sample entropy; ANOVA, analysis of variance

## Discussion

In alignment with our primary hypothesis, this study demonstrates for the first time, that an intensive 12-week BMT program increased vagally mediated RMSSD and slowed HR during overnight sleep. Exploratory analyses of less-established HRV metrics collected from the frequency domain (e.g., HF power and the LF:HF ratio) provided further evidence of increased vagal activity. These changes, typically observed alongside enhanced cardiorespiratory fitness (Goldsmith et al. 1992), were still evident despite the high allostatic stress experienced during BMT (Drain et al. 2017). However, in this apparently healthy cohort, no correlation was evident between fasted morning circulating TNFα concentrations, which also reduced over time, and RMSSD, a HRV estimate of cardiac vagal autonomic modulation (Task Force 1996). The unique observations from this study indicate that the BMT program provides suitable physical and cognitive stressors that, at a group level, are strong enough to promote increases in vagally mediated RMSSD and slow overnight sleeping HR whilst also conveying a small yet vagally independent reduction of TNFα, reflecting lowered inflammatory status.

The primary outcome from the current study was the progressive increases in the well-established vagally mediated HRV metric of RMSSD throughout 12 weeks of BMT in these young and healthy participants. In alignment, HF power, a frequency domain metric reflective of vagal activity, was significantly increased in week 12 and predominantly responsible for driving the observed reduction in the LF:HF power ratio. This observation contrasts with findings from previous studies demonstrating that in otherwise healthy individuals, prolonged high-volume training demands and/or additional stressors, including compromised sleep, insufficient recovery, and fatigue, are associated with mixed HRV outcomes (Meeusen et al. 2013; Westphal et al. 2021; Pichot et al. 2002). The differing results may be explained by the volume and intensity of the physical training prescribed which significantly increased cardiorespiratory fitness by 7% at week 8. Further, although the novel BMT program that recruits completed in the current study includes training activities that are physically and cognitively challenging, it primarily prescribes low-moderate intensity physical activity with intermittent periods of high-intensity exercise (Burley et al. 2020), whereas traditional military physical training programs have emphasised high-volume cardiorespiratory and muscular endurance activities (Kyröläinen et al. 2018). Nevertheless, Grant et al. (2016) and Jouanin et al. (2004) demonstrated similar effects on short-term (5 min) awake recordings of HRV metrics reflecting cardiac vagal modulation; we support and strengthen these observations by monitoring during prescribed overnight sleep opportunities incorporating the nadir HR.

In line with the primary HRV outcome of the study, on average a progressive 8 b min^−1^ slowing of overnight sleeping HR was observed at week 12 of BMT. This 12% reduction is consistent with typical effects of endurance training on heart rhythm between an ‘untrained’ and a ‘trained’ group (Goldsmith et al. 1992). However, there were a small number of recruits amongst the cohort in which sleeping HR remained stable or slightly increased (Fig. 1b). Similar observations have been made previously, whereby nocturnal HRV increased in most but not all individuals subjected to four weeks of endurance training (Nummela et al. 2016). It is likely the non-response is explained, at least in part by a higher training status prior to commencing BMT (Pickering and Kiely 2019), individual fluctuation of HR and HRV related to sleep or other overnight disturbance (Hinde et al. 2021), or human variance in response to exercise training and highlights the importance of future studies considering precision exercise medicine to promote uniform responses (Ross et al. 2019). Nonetheless, slowing of resting HR is most commonly attributed to increased vagal tone, particularly in studies investigating exercise training induced resting bradycardia (Coote and White 2015). However, the decline observed in resting HR could be explained by additional contributory factors, other than exclusively a change in vagal tone. Indeed, direct intrinsic adaptations within myocardial tissue are known to promote a resting bradycardia (Boyett et al. 2017). Our results with respect to entropy and RMSSD (corrected for underlying HR) support the notion that intrinsic factors may have also contributed to the decline in resting HR (Boyett et al. 2013). Entropy is a measure of the degree of irregularity or the likelihood that successive RR intervals will repeat themselves, with greater values thought to indicate increased complexity of the physiological processes underlying the control of HR (Pincus and Goldberger 1994). We observed in our investigation a significantly increased entropy at week 8 of BMT. Similarly, with respect to RMSSD corrected for underlying HR, we demonstrated that vagally mediated RMSSD was proportionally decreased at each time point. Whilst debate exists as to whether HRV is a valid measure of cardiac autonomic modulation or is primarily a non-linear surrogate of HR itself (Boyett et al. 2019; Malik et al. 2019; Macartney et al. 2021), these results suggest progressive and proportional reductions in vagal modulation when RMSSD is normalised to HR. When taken together, the HR corrected results in combination with measures of entropy appear to support the notion of direct intrinsic adaptations of the cardiac tissue contributing towards exercise induced resting bradycardia (Boyett et al. 2013). Thus, an adaptive interplay of neural (vagal) modulation and structural changes to cardiac tissue may collectively be responsible for the observed decline in HR; however, the answer to this question is outside the scope of the current study. Irrespective of mechanism, the observed reduction in HR during extended periods of sleep in these recruits conveys an ivabradine like effect on coronary artery perfusion (increased) which occurs primarily during diastole (Heusch 2008).

Interestingly, in the current study, despite significantly reduced mean circulating TNFα concentrations at week 12 and progressive increases in RMSSD, no correlations between these measures were observed at any of the time points during BMT. There are several possible reasons for this. Firstly, chronic systemic inflammation is predominantly observed in individuals pushed routinely to the limits of their physical capabilities combined with insufficient recovery (Carfagno and Hendrix 2014). It is unlikely that the stressors within the BMT program are strenuous enough over the 12 weeks to stimulate such an inflammatory response. In fact, the BMT program has recently been demonstrated to cause minimal perturbation to inflammatory markers (Tait et al. 2021). Second, RMSSD is a surrogate measure of vagal activity upon sino-atrial nodal tissue (Gourine and Ackland 2019). Accordingly, increases in RMSSD may not directly translate to increased vagal nerve activity by the multi-synaptic interaction between the vagus and the splanchnic nerves (Bassi et al. 2020) modulating splenic macrophage TNFα release (Bonaz et al. 2016; Alen et al. 2021). Finally, high inflammatory states are commonly observed in older (Alen et al. 2021) or cardiometabolic chronic disease cohorts with elevated resting HR (Osborn and Olefsky 2012). In combination, these data lead us to reject the postulation that an increase in HRV, as a reflection of cardiac specific vagal nerve activity, may be associated with indirect inhibition of the release of circulating TNFα in young and healthy individuals.

The reduction observed in this study of overnight sleeping DFAα1 away from 1.0 was also noteworthy. This metric of fractal complexity is quantified using values from 0.5 to 1.5, with lower values indicating more random and higher values indicating more correlated interbeat variability behaviour, and a value of approximately 1.0 signifying a balance between random and predictable (self-similar) interbeat behaviour (Peng et al. 1995). In healthy individuals, relative to their awake measurements, DFAα1 has been demonstrated to be increased during REM sleep but reduced during light sleep with a further reduction during deep sleep (Penzel et al. 2003; Bunde et al. 2000). Accordingly, the lower sleeping DFAα1 values observed during week 8 and 12 within the current study may well indicate a shift in sleep patterns towards predominantly deep sleep. However, this point is rather speculative as it has been acknowledged that further work is needed to understand the link between sleep stage and heart rhythm (Penzel et al. 2003).

In conclusion, the current study reveals that 12-weeks of BMT in Army recruits increases vagally mediated RMSSD and slows HR during overnight sleep with an eventual reduction on circulating TNFα concentrations. These results indicate that, on average, recruits are achieving cardiovascular and inflammatory health benefits despite experiencing cumulative stressors associated with the Australian Army 12-week BMT course. However, it was evident that a small number of individuals did not respond in alignment with the group and further research is necessary to identify the potential contributing factors (e.g., nutrition, hydration, psychological) at an individual level. This highlights the need to engage further with understanding human physiological variation (Tipton et al. 2021) in response to multi-stressor environments such as BMT. Nevertheless, the homogenous young and healthy group used in the study, alongside prescribed overnight sleep opportunities and the tightly controlled nature of a military training environment make these findings particularly novel relative to other less controlled applied settings. Lower resting HR and increased HRV are strongly associated with superior exercise capacity (Gourine and Ackland 2019), maintenance of which is essential for well-being and optimal cardiac health outcomes across a lifespan, and for promoting career longevity in military recruits.

## Data Availability

Data will be made available upon request to the corresponding author.

## Abbreviations

ANOVA: Analysis of variance
ApEn: Approximate entropy
b min^−^^1^: Beats per minute
BMT: Basic military training
DFA: Detrended fluctuation analysis
HF: High frequency
HR: Heart rate
HRV: Heart rate variability
LF: Low frequency
ms: Milliseconds
RMSSD: Root mean square of successive differences
SampEn: Sample entropy
TNFα: Tumor necrosis factor alpha
VO_2_max: Maximal oxygen consumption
